# Cerebral autoregulation testing in a porcine model of intravenously administrated *E. coli *induced fulminant sepsis

**DOI:** 10.1186/cc14081

**Published:** 2015-03-16

**Authors:** L Molnar, M Berhes, L Papp, N Nemeth, B Fulesdi

**Affiliations:** 1 Deoec Aneszteziologia, Debrecen, Hungary; 2 University of Debrecen, Hungary

## Introduction

To assess cerebral hemodynamics in an experimental sepsis model.

## Methods

Nineteen juvenile female Hungahib pigs were subjected into control group (*n *= 9) or septic group (*n *= 10). Under general anesthesia in animals of the sepsis group, *Escherichia coli *culture (2.5 × 10^5^/ml; strain: ATCC 25922) was intravenously administrated in a continuously increasing manner as follows: 2 ml in the first 30 minutes, then 4 ml in 30 minutes and afterwards 16 ml/hour for 2 hours (so a total of 9.5 × 10^6 ^*E. coli *within 3 hours). In the control group the anesthesia was maintained for 8 hours, infusion was administered as a similar volume of isotonic saline solution and no other intervention was made. Hemodynamic monitoring of all animals was performed by PiCCo monitoring system. The middle cerebral artery of the pigs was insonated through the transorbital window and cerebral blood flow velocity (MCAV) and pulsatility index was registered.

## Results

In the septic group, as expected, all animals developed fulminant sepsis and died within 3 to 7 hours two animals in 3 to 4 hours, and three in 6 to 7 hours). In the septic animals the heart rate rose and mean arterial pressure dropped, their ratio increased significantly compared with both the base values (at the 6th hour: *P *< 0.001) and the control group (*P *= 0.004). The control animals showed stable condition over the 8-hour anesthesia. MCAV significantly decreased during the development of sepsis (from 23.6 ± 6.6 cm/s to 16.0 ± 3.9 cm/s, *P *< 0.01) and pulsatility indices increased (from 0.68 ± 0.22 to 1.37 ± 0.58, *P *< 0.01), indicating vasoconstriction of the resistance vessels. A significant relationship was fund between percent change of the MAP and the pulsatility index in septic animals (*R*^2 ^= 0.32) referring to maintained cerebral autoregulation.

## Conclusion

Cerebral autoregulation is preserved in the pig model of experimentally induced fulminant sepsis.

